# Gut Microbiota Dysbiosis after Traumatic Brain Injury Contributes to Persistent Microglial Activation Associated with Upregulated Lyz2 and Shifted Tryptophan Metabolic Phenotype

**DOI:** 10.3390/nu14173467

**Published:** 2022-08-24

**Authors:** Zhipeng Zheng, Shuai Wang, Chenghao Wu, Yang Cao, Qiao Gu, Ying Zhu, Wei Zhang, Wei Hu

**Affiliations:** 1 Department of Critical Care Medicine, Fourth Clinical Medical College of Zhejiang Chinese Medical University, Hangzhou 310006, China; 2 Department of Critical Care Medicine, Affiliated Hangzhou First People’s Hospital, Zhejiang University School of Medicine, Hangzhou 310006, China; 3 Department of Neurosurgery, Affiliated Hangzhou First People’s Hospital, Zhejiang University School of Medicine, Hangzhou, 310006 China; 4 Department of General Surgery, Secondary Clinical Medical College of Zhejiang Chinese Medical University, Hangzhou 310005, China

**Keywords:** gut microbiota, tryptophan metabolism, microglia, Lyz2, chronic neuroinflammation, traumatic brain injury

## Abstract

Traumatic brain injury (TBI) is a common cause of disability and mortality, affecting millions of people every year. The neuroinflammation and immune response post-TBI initially have neuroprotective and reparative effects, but prolonged neuroinflammation leads to secondary injury and increases the risk of chronic neurodegenerative diseases. Persistent microglial activation plays a critical role in chronic neuroinflammation post-TBI. Given the bidirectional communication along the brain–gut axis, it is plausible to suppose that gut microbiota dysbiosis post-TBI influences microglial activation. In the present study, hippocampal microglial activation was observed at 7 days and 28 days post-TBI. However, in TBI mice with a depletion of gut microbiota, microglia were activated at 7 days post-TBI, but not at 28 days post-TBI, indicating that gut microbiota contributes to the long-term activation of microglia post-TBI. In addition, in conventional mice colonized by the gut microbiota of TBI mice using fecal microbiota transplant (FMT), microglial activation was observed at 28 days post-TBI, but not at 7 days post-TBI, supporting the role of gut microbiota dysbiosis in persistent microglial activation post-TBI. The RNA sequencing of the hippocampus identified a microglial activation gene, Lyz2, which kept upregulation post-TBI. This persistent upregulation was inhibited by oral antibiotics and partly induced by FMT. 16s rRNA gene sequencing showed that the composition and function of gut microbiota shifted over time post-TBI with progressive dysbiosis, and untargeted metabolomics profiling revealed that the tryptophan metabolic phenotype was differently reshaped at 7 days and 28 days post-TBI, which may play a role in the persistent upregulation of Lyz2 and the activation of microglia. This study implicates that gut microbiota and Lyz2 are potential targets for the development of novel strategies to address persistent microglial activation and chronic neuroinflammation post-TBI, and further investigations are warranted to elucidate the specific mechanism.

## 1. Introduction

Traumatic brain injury (TBI), caused by an external force to the head resulting in a skull fracture, brain contusion, or intracranial hematoma, is a common cause of death and disability [1]. Almost half of the world’s population is likely to experience TBI at least once in their lifetime [2]. Worldwide, more than 50 million people are affected by TBI each year, with 10 million dead and nearly 30 million permanently disabled, and TBI survivors suffer from physical, psychological, and cognitive impairments, which burden families and society [1]. The acute immune response and neuroinflammation post-TBI involves recruitment of peripheral immune cells, changes in microglia, astrocytes, oligodendrocytes, and endothelial cells, and the production of pro-inflammatory and anti-inflammatory cytokines and chemokines, which initially have neuroprotective and reparative effects, but prolongations of these processes lead to neurodegeneration [3]. Although the advanced progress of brain injury study and technology has significantly improved the clinical outcome of TBI patients in recent decades, the neuroinflammatory response in acute and chronic periods post-TBI may aggravate the primary injury and lead to neurodegenerative disorders, which lacks effective treatment and the underlying mechanism is still not fully understood [4].

Microglia, the major cellular components of the innate immune in the brain, play a critical role in the production of neuroprotective factors, clearance of cellular debris, and orchestration of neurorestorative processes, which promote neurological recovery post-TBI [5]. However, the prolonged overactivation of microglia can produce excessive pro-inflammatory and cytotoxic mediators that impede central nervous system (CNS) repair, lead to neuronal dysfunction, and increases the risk of neurodegenerative disease [6]. Recent studies have reported that microglial elimination followed by recolonization can improve the outcomes of TBI in mice, and similar approaches also have beneficial effects in mice with autism or Alzheimer’s disease (AD) [7]. Therefore, it is necessary to reveal the mechanism of persistent microglial activation for weeks, months, or even years after the primary brain injury post-TBI to prevent or inhibit chronic neuroinflammation.

A growing body of clinical and preclinical research has evidenced that gut microbiota has a crucial role in key aspects of neurodevelopment, neuroinflammation, and behavior [8]. The microbiota–gut–brain axis, a set of bidirectional communication pathways between gut microbiota and the CNS, has emerged as an exciting research area in neurology. The dysfunction of this axis contributes to the pathogenesis of neurological diseases, such as multiple sclerosis, autism spectrum disorder, Parkinson’s disease (PD), AD, stroke, and brain injury [8]. Gut microbiota, gut-derived neurotransmitters, and gut barrier function are all involved in the regulation of the CNS. In TBI, the primary damage directly activates microglia and impairs intestinal mucosa, leading to changes in gut microbiota composition and gut barrier function, which further triggers systemic immune response [9]. Peripheral pro-inflammatory cytokines influence the CNS and act on the microglia that are previously primed from the local brain injury, mediating persistent neuroinflammation [9]. Although preclinical data suggest that gut microbiota is involved in this bidirectional process of TBI, the complex role of the gut microbiota in the long-term microglial activation post-TBI deserves further study.

Gut microbiota regulates the proliferation, maturation, and function of microglia; mechanically, short-chain fatty acids (SCFAs) produced by gut microbiota promote microglia maturation, and the tryptophan metabolites of gut microbiota influence microglial functions through aryl hydrocarbon receptor (AhR) [10,11]. It is worth noting that tryptophan is under the co-metabolism of gut microbiota and the host. The three pathways of tryptophan metabolism, including the indole pathway, serotonin pathway, and kynurenine pathway, are directly or indirectly modulated by gut microbiota, which plays an important role in disease and health [12]. In addition, previous research on TBI has found that tryptophan is differentially metabolized in astrocytes and microglia, producing neuroprotective kynurenic acid and neurotoxic quinolinic acid, respectively [13]. Nevertheless, it is unclear whether changes in gut microbiota and its tryptophan metabolism have an effect on microglial activation and neuroinflammation post-TBI, and the underlying mechanism needs to be further studied.

## 2. Methods

### 2.1. Animal Experiment

C57BL/6 male mice (4–6 weeks old, 16–18 g) were purchased from Shanghai SLRC Laboratory Animals Co., Ltd. (Shanghai, China). All mice were kept in a temperature and humidity-controlled specific pathogen-free (SPF) facility with a light–dark cycle (12 h:12 h, light on at 6:00). All mice were free to access a standard laboratory diet. After two weeks of acclimatization, the mice were randomly divided into groups. Fourteen days before traumatic brain injury (TBI), the mice of group A began to be treated with broad-spectrum antibiotics (Abx) by gavage once per day. On day 0, the mice of group T and A were subjected to TBI, whereas the mice of group C were subjected to a sham operation. One day after TBI, the mice of group F began to receive fecal microbiota transplant (FMT) using feces collected from group T once per day. Seven days after TBI, half the mice of each group were sacrificed for sample collection. Twenty-eight days after TBI, the remaining mice of each group were sacrificed for sample collection. The protocols of animal experiments were under the guidelines for the care and use of laboratory animals of the National Institutes of Health and approved by the Animal Care Ethics Committee of Zhejiang Chinese Medical University Laboratory Animal Research Center.

### 2.2. Depletion of Gut Microbiota

For the depletion of gut microbiota, each mouse of Group A was gavaged with 300 µL sterile distilled water containing an antibiotic cocktail: 0.96 mg vancomycin hydrochloride, 1.86 mg neomycin sulfate, 1.86 mg ampicillin sodium salt, and 1.20 mg metronidazole. These antibiotics could effectively deplete gut bacteria and were used to model a germ-free condition in previous reports [14].

### 2.3. Traumatic Brain Injury Model 

A controlled cortical impact (CCI) surgery was performed to induce TBI, as previously described [15]. Briefly, the mice of Group T and A were anesthetized by the intraperitoneal injection of pentobarbital sodium (0.05 mg/g) and placed in a stereotaxic frame. A middling incision was made, the skin was retracted, and a 4-mm-diameter craniotomy was performed at a point midway between the lambda and bregma sutures, over the right parietal cortex, 2 mm lateral to the midline, using a portable drill. The skull cap was carefully removed, and the underlying leptomeninges were kept intact. A 2.5-mm-diameter impact tip was kept perpendicular to the exposed cortical surface using a PinPoint™ Precision Cortical Impactor (Cary, NC, USA). The parameter settings for the CCI were as follows: impact speed, 3.0 m/s; deformation depth, 1.0 mm; duration, 100 ms. After surgery, the incision was sutured, and the mice were placed on a warming pad to maintain core body temperature at 37 ± 0.5 °C until recovering from anesthesia.

### 2.4. Fecal Microbiota Transplant

A fecal microbiota transplant (FMT) was performed according to the modified method described previously [16]. In short, fresh fecal pellets of TBI mouse were collected daily, mixed, and homogenized in sterile PBS (150 mg/mL), and 200 µL of the fecal suspension was administered to a recipient mouse of Group F via oral gavage once a day for 1 week or 4 weeks. To reduce the commensal gut microbiota, each recipient mouse was provided with 300 µL of sterile distilled water containing 0.96 mg of vancomycin hydrochloride, 1.86 mg of neomycin sulfate, 1.86 mg of ampicillin sodium salt, and 1.20 mg of metronidazole via oral gavage once a day for 3 days, and then washed out for 3 days before FMT.

### 2.5. Histology

Freshly harvested brain tissues were immediately fixed in 4% paraformaldehyde overnight, and subsequently dehydrated and embedded in paraffin. Paraffin-embedded brain tissues were coronally sectioned along the injury site at a thickness of 4 μm, used for hematoxylin and eosin (H&E) staining. The stained brain tissue slices were examined using a biomicroscope.

### 2.6. Immunofluorescence Examination

For ionized calcium-binding adaptor molecule 1 (IBA-1) immunofluorescence staining, the paraffin-embedded brain tissues were coronally sectioned along the injury site at a thickness of 4 μm. After antigen retrieval, the brain sections were incubated with anti-IBA-1 rabbit antibody (Servicebio, Wuhan, China) overnight at 4 °C, and then incubated with Cy3-conjugated goat anti-rabbit antibody (Servicebio, Wuhan, China) for 50 min at room temperature. DAPI (Servicebio, Wuhan, China) was used to stain the nuclei. The stained images of brain tissue slices were captured by a fluorescence microscope (Eclipse C1; Nikon, Japan).

### 2.7. RNA Sequencing

The total RNA extracted from hippocampus tissue was used to synthesize cDNA for library construction. RNA sequencing was conducted on the DNBSEQ platform (BGI, Wuhan, China). Data processing was performed using an online platform provided by BGI (https://biosys.bgi.com) accessed on 30 March 2022.

### 2.8. Untargeted Metabolomics Profiling

Serum was extracted by centrifuging the blood samples at 4000 rpm for 10 min, and then stored at −80 °C until analysis. Metabolite extraction was performed according to previously reported methods [17]. In brief, 50 μL of serum was mixed with 150 μL of precooling methanol (MeOH)/acetonitrile (2:1 (v/v)). After vortexing for 1 min and then static placing at –20 °C for 2 h, the mix was centrifuged for 20 min (4 °C, 4000 rpm). Then, 150 μL of the supernatant was transferred for vacuum freeze drying and resuspended in 75 μL of MeOH/water (1:1 (v/v). The mix was centrifuged for 30 min (4 °C, 4 000 rpm) and the supernatant was used for analysis. Additionally, a procedural blank was used to monitor contamination, and a pooled quality control (QC) sample was prepared by mixing 10 μL of the final supernatant of each sample.

The samples were analyzed on an ultra-performance liquid chromatography (UPLC) tandem mass spectrometry (MS) system with a heated electrospray ionization (HESI) source and controlled by the Xcalibur 2.3 software program (Thermo Fisher Scientific, Waltham, MA, USA). UPLC was performed on a Waters 2D UPLC system with a ACQUITY UPLC BEH C18 analytical column (1.7 µm, 2.1 mm × 100 mm; Waters, Milford, MA, USA) at 45 °C. Under electrospray ionization-positive (ESI+) mode, mobile phase A was water with 0.1% formic acid (v/v) and mobile phase B was MeOH with 0.1% formic acid (v/v). Under electrospray ionization-negative (ESI–) mode, mobile phase A was water with 10 mM ammonium formate and mobile phase B was 95% MeOH with 10 mM ammonium formate. When processing a sample, the optimized linear elution gradient was as follows: the composition of mobile phase B was 2% in 1 min, increased to 98% in the next 8 min, kept for 3 min, and then decreased to 2% in 0.1 min and kept for 2.9 min before the run stop. The injection volume was 5 μL and the flow rate was 350 μL/min.

A Q Exactive HF MS (Thermo Scientific, Waltham, MA, USA) was used to perform the MS under both ESI+ and ESI– mode. The MS spray voltages were 3.8 kV under ESI+ mode and 3.2 kV under ESI– mode. The capillary temperature was set at 320 °C with the sheath gas at 40 arbitrary units (AUs), the auxiliary gas at 10 AUs, and the sweep gas at 2 AUs. The acquisition mode was full MS and the scan range was from 70 to 1050 m/z. The resolution was set at 120,000 for the full MS and 30,000 for the MS/MS. The MS/MS analysis was performed with the collision energy at 20, 40, and 60 eV.

The samples were randomly ordered to reduce system errors and a QC sample was interspersed for every 10 samples. The raw data were initially handled by Compound Discoverer 3.1 software (Thermo Fisher Scientific, Waltham, MA, USA). BMDB database, mzCloud, and ChemSpider (HMDB, KEGG, LipidMaps) databases were used for the identification of metabolites.

### 2.9. 16s rRNA Gene Sequence

The total bacterial DNA extracted from snap-frozen colon contents was used for sequencing the V3 and V4 regions of the 16s rRNA gene on an Illumina HiSeq platform (Illumina, San Diego, CA, USA). The analysis of the sequencing data was conducted using the Quantitative Insights into Microbial Ecology (QIIME) software package (v2.0, Flagstaff, AZ, USA). All reads were assigned to operational taxonomic units (OTUs) with 97% sequence identity and taxonomically classified using the Greengene reference database and Usearch software (v7.0.1090). The α-diversity was measured using Chao, Ace, Shannon, and Simpson indexes. The β-diversity was evaluated using the weighted UniFrac distance matrix to conduct the principal coordinates analysis (PCoA). The linear discriminant analysis (LDA) effect size (LEfSe) method was used to identify the taxa of different bacteria with statistical significance. The Phylogenetic Investigation of Communities by Reconstruction of Unobserved States 2 (PICRUSt2) was used to infer the predicted function of the gut microbiota.

### 2.10. Statistical Analysis

Statistical analyses and graph preparation were performed by GraphPad Prism 9.0 (GraphPad, La Jolla, CA, USA). Bar graphs were shown as the mean ± standard deviation (SD) or mean with a range. The significance between the two groups was determined by an unpaired Student’s t-test or Wilcoxon rank-sum test. A *p*-value < 0.05 was considered statistically significant.

## 3. Results

### 3.1. Gut Microbiota Dysbiosis Mediated Prolonged Microglial Activation in the Hippocampus of Mice with TBI

**Figure 1:**
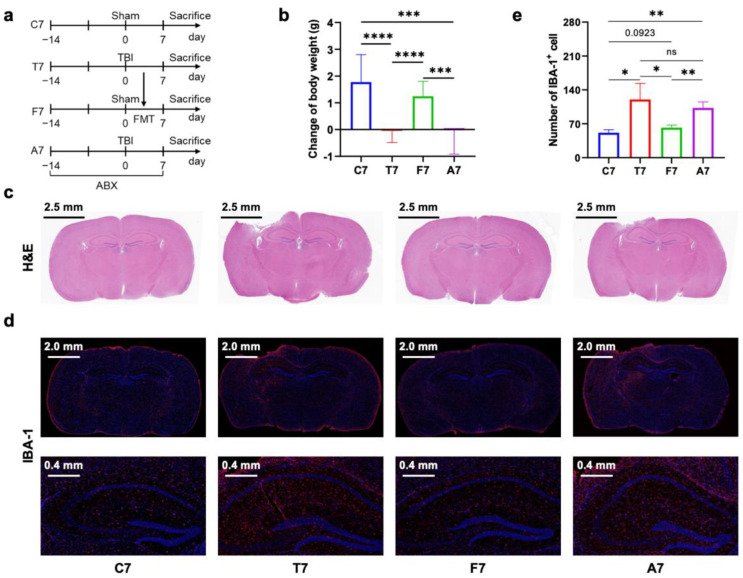
Design of the experiment and evaluation of brain injury and microglial activation at 7 days postTBI. (**a**) Design of the experiment (*n* = 6 per group). Mice of group C7 received sham surgery, mice of group T7 were subjected to TBI surgery without perioperative antibiotic treatment, the mice of group F7 underwent sham surgery and FMT using fecal microbiota from the mice of group T7, and mice of group A7 were subjected to TBI surgery with perioperative antibiotic treatment. All mice were sacrificed 7 days after TBI for sample collection. (**b**) Change of body weight before and after TBI (*n* = 6 per group). (**c**) Representative images of the brain H&E staining (*n* = 3 per group). (**d**) Representative images of brain IBA-1 immunofluorescence staining (red) (*n* = 3 per group). (**e**) Number of IBA-1 positive cells in hippocampus. Data are shown as mean ± SD. * *p* < 0.05, ** *p* < 0.01, *** *p* < 0.001, **** *p* < 0.0001 using unpaired Student’s *t* test. TBI, traumatic brain injury; H&E, hematoxylin–eosin; IBA-1, ionized calcium binding adapter molecule 1; ns, no significant.

**Figure 2:**
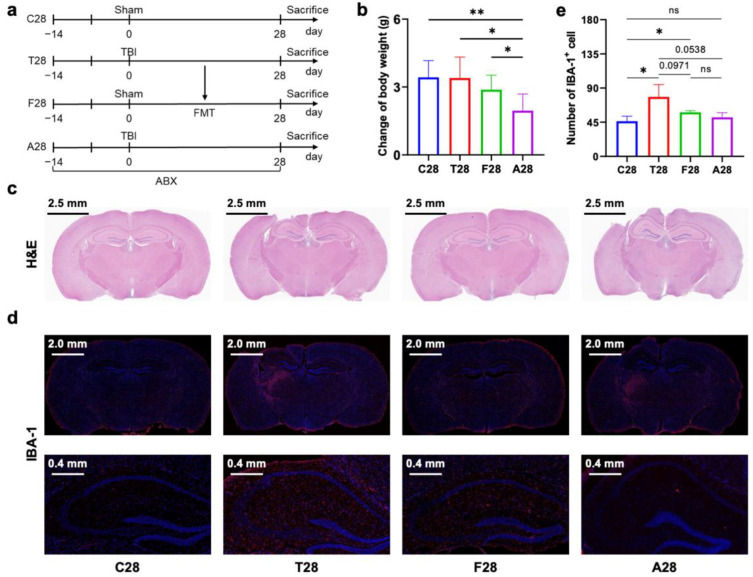
Design of the experiment and evaluation of brain injury and microglial activation at 28 days post-TBI. (**a**) Design of the experiment (*n* = 6 per group). Mice of group C28 received sham surgery, mice of group T28 were subjected to TBI surgery without perioperative antibiotic treatment, mice of group F28 underwent sham surgery and FMT using fecal microbiota from the mice of group T28, and the mice of group A28 were subjected to TBI surgery with perioperative antibiotic treatment. All mice were sacrificed 28 days after TBI for sample collection. (**b**) Change of body weight before and after TBI (*n* = 6 per group). (**c**) Representative images of brain H&E staining (*n* = 3 per group). (**d**) Representative images of brain IBA-1 immunofluorescence staining (red) (*n* = 3 per group). (**e**) The number of IBA-1 positive cells in the hippocampus. Data are shown as mean ± SD. * *p* < 0.05, ** *p* < 0.01 using unpaired Student’s *t* test. TBI, traumatic brain injury; H&E, hematoxylin–eosin; IBA-1, ionized calcium-binding adapter molecule 1; ns, no significant.

Given our hypothesis that gut microbiota dysbiosis post-TBI contributed to chronic neuroinflammation in the brain, we investigated the effects of oral broad-spectrum antibiotics in TBI mice and the effects of FMT (feces collected from TBI mice) in normal mice (Figure 1a and Figure 2a). Two time points of observation (7 days and 28 days post-TBI) were established according to previous studies wherein activated microglia appeared a bimodal increase with an initial peak at 7 days followed by a secondary peak at 21–28 days after focal brain contusion [6]. After 7 days, TBI with or without depletion of gut microbiota reduced body weight gain (Figure 1b). However, after 28 days, only TBI with depletion of gut microbiota reduced body weight gain (Figure 2b), suggesting that gut microbiota has a role in the long-term maintenance of body weight post-TBI. Additionally, FMT did not affect body weight. H&E stained coronal sections showed a local cortical lesion in the right hemisphere of mice with TBI (Figure 1c and Figure 2c). IBA-1, a marker of microglial activation and proliferation [18], and immunofluorescence-stained sections were used to assess microglial activation post-TBI (Figure 1d and Figure 2d). By quantifying the number of IBA-1 positive cells in the ipsilateral hippocampus, TBI mice without the depletion of gut microbiota had an increase in microglial activation at 7 days and 28 days post-TBI, whereas TBI mice with the depletion of gut microbiota only had an increase in microglial activation at 7 days post-TBI but not at 28 days post-TBI (Figure 1e and Figure 2e), indicating that gut microbiota dysbiosis post-TBI mediates long-term microglia activation. In addition, FMT derived from TBI mice to normal mice for 28 days but not for 7 days increased microglia activation (Figure 1e and Figure 2e), which further supported that long-term gut microbiota dysbiosis post-TBI contributed to prolonged microglial activation in the hippocampus.

### 3.2. Gut Microbiota-Dependent Persistent Upregulation of Lyz2 Post-TBI Was Parallel with Prolonged Microglial Activation

**Figure 3:**
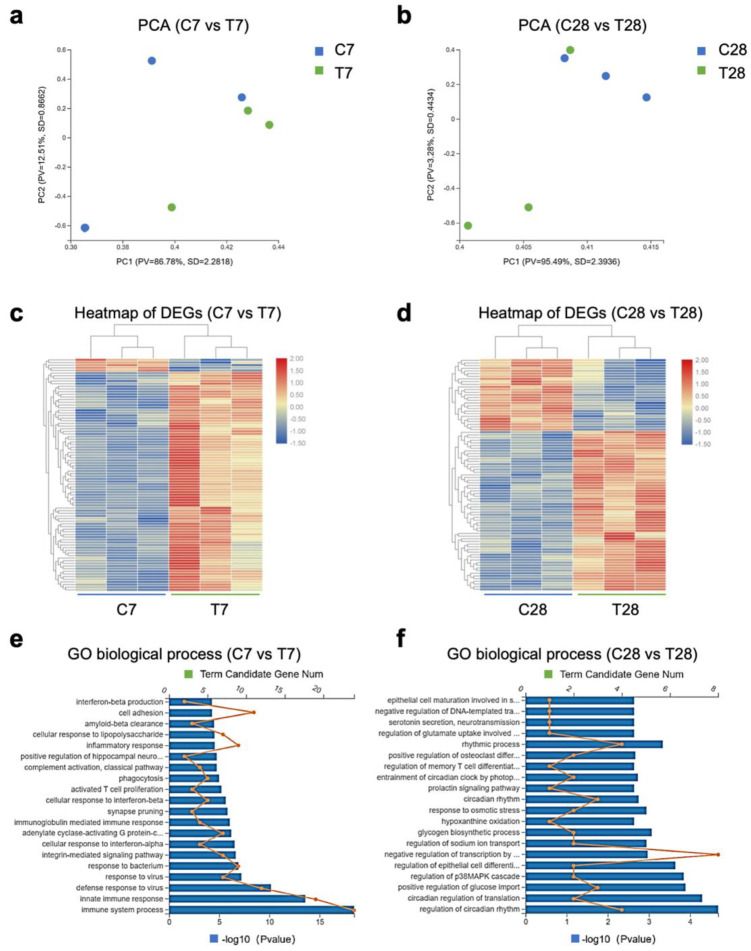
Transcriptome analysis of hippocampus post-TBI. PCA of the hippocampus RNA sequencing data at 7 days (**a**) and 28 days (**b**) post-TBI (*n* = 3 per group). Heatmap of DEGs in the hippocampus at 7 days (**c**) and 28 days (**d**) post-TBI (*n* = 3 per group). GO biological process enrichment analysis of DEGs in the hippocampus at 7 days (**e**) and 28 days (**f**) post-TBI (*n* = 3 per group). TBI, traumatic brain injury; PCA, principal component analysis; DEGs, differential expressed genes; GO, gene ontology.

To uncover the mechanism of persistent microglial activation in the hippocampus post-TBI, RNA sequencing was conducted to study the changes in hippocampal gene expression. Principal component analysis (PCA) showed that hippocampal gene expressions at 7 days and 28 days post-TBI were quite distinct from those of control mice (Figure 3a,b). Heatmap of differentially expressed genes (DEGs) between control mice and TBI mice indicated that hippocampal genes were differentially expressed at 7 days and 28 days post-TBI (Figure 3c,d). A gene ontology (GO) biological process enrichment analysis of the DEGs displayed the top 20 biological processes with the most significant difference, which had big changes at 7 days and 28 days post-TBI (Figure 3e,d). Specifically, synapse pruning significantly changed at 7 days post-TBI, while several circadian-associated biological processes showed changes at 28 days post-TBI.

**Figure 4:**
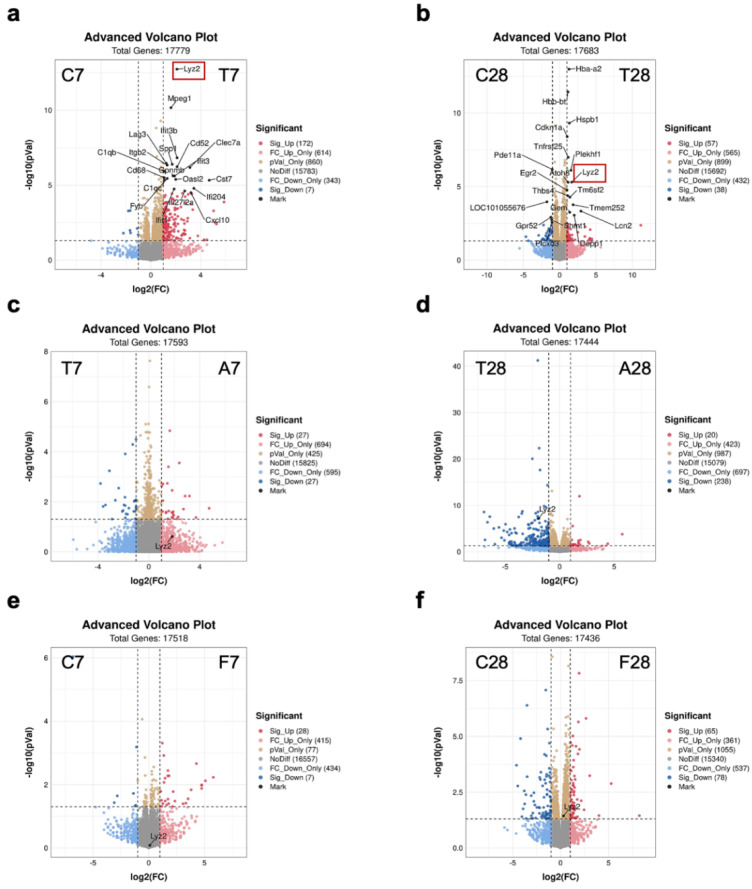
Gut microbiota-dependent persistent upregulation of Lyz2. Advanced volcano plot analysis of gene expression levels of the hippocampus: between control mice and TBI mice at 7 days (**a**) and 28 days (**b**) post-TBI; between TBI mice and antibiotic-treated TBI mice at 7 days (**c**) 28 days (**d**) post-TBI; between control mice and TBI-colonized mice at 7 days (**e**) and 28 days (**f**) post-TBI (*n* = 3 per group). TBI, traumatic brain injury; Lyz2, lysozyme 2.

Furthermore, advanced volcano plots marked with the top 20 DEGs between control mice and TBI mice were made to identify specific DEGs that may contribute to long-term microglia activation. Notably, compared to control mice, Lyz2 was significantly upregulated at 7 days post-TBI (Figure 4a), and among the top 20 DEGs, the only one gene still in a state of upregulation 28 days after TBI (Figure 4b). More importantly, Lyz2 is a microglial activation gene [19]. Interestingly, compared to the antibiotic-treated TBI mice with gut microbiota depletion, Lyz2 was not upregulated in TBI mice at 7 days post-TBI (Figure 4c) but significantly upregulated in TBI mice at 28 days post-TBI (Figure 4d), suggesting that persistent Lyz2 upregulation 28 days after TBI was dependent on gut microbiota. Additionally, FMT from TBI mice to normal mice for 7 days did not affect Lyz2 expression (Figure 4e), but FMT for 28 days upregulated Lyz2 to a certain extent (Figure 4f). This effect of gut microbiota of TBI mice on normal mice further supported that constant changes in gut microbiota after TBI contributed to the persistent upregulation of Lyz2, which was associated with prolonged microglia activation and chronic neuroinflammation.

### 3.3. Tryptophan Metabolic Phenotype Was Differently Reshaped at 7 Days and 28 Days Post-TBI

**Figure 5:**
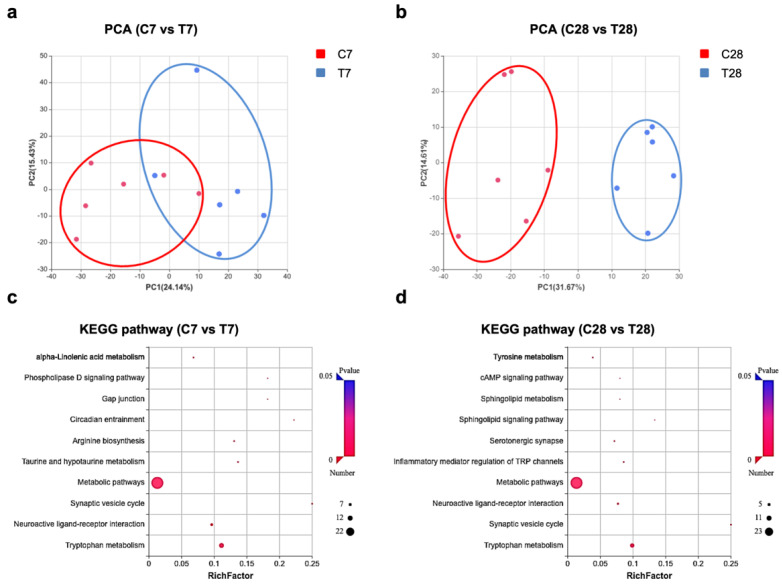
Untargeted metabolome analysis of serum post-TBI. PCA of the serum untargeted metabolic data at 7 days (**a**) and 28 days (**b**) post-TBI (*n*= 6 per group). KEGG pathway enrichment analysis of differential metabolites in serum at 7 days (**c**) and 28 days (**d**) post-TBI (*n* = 6 per group). TBI, traumatic brain injury; PCA, principal component analysis; KEGG, Kyoto Encyclopedia of Genes and Genomes.

Given that the role of gut microbiota in host health and disease is often mediated by its metabolites [20], and the blood–brain barrier (BBB) has persistent dysfunction post-TBI [3], global metabolic profiling was performed to detect serum metabolites. PCA showed that changes in serum metabolites were more pronounced at 28 days post-TBI (Figure 5b), compared to that at 7 days post-TBI (Figure 5a). A Kyoto Encyclopedia of Genes and Genomes (KEGG) enrichment analysis of the differential metabolites between control mice and TBI mice displayed the top 10 pathways with the most significant difference (Figure 5c,d). Significantly, tryptophan metabolism, neuroactive ligand–receptor interaction, synaptic vesicle cycles, and metabolic pathways changed at 7 days as well as 28 days post-TBI. Additionally, serotonergic synapse associated with tryptophan metabolism only changed at 28 days post-TBI.

**Figure 6:**
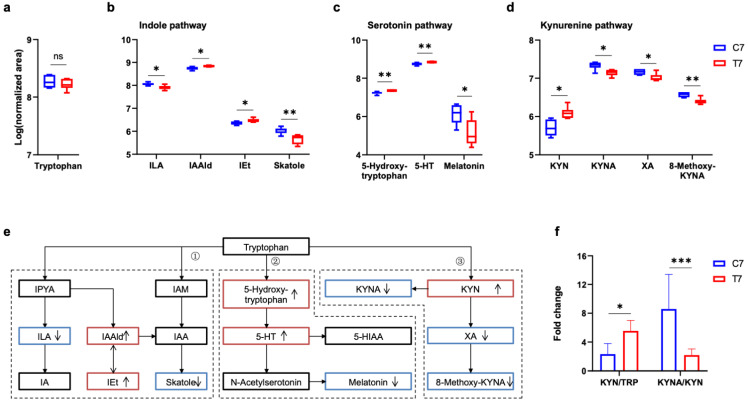
Alterations of the serum tryptophan metabolic pathway at 7 days post-TBI. Comparison of serum tryptophan (**a**) and major tryptophan metabolites in the indole pathway (**b**), serotonin pathway (**c**), and kynurenine pathway (**d**) at 7 days post-TBI. (**e**) Major tryptophan metabolism pathways are involved: (1) Indole pathway: Tryptophan can be directly metabolized by the gut microbiota into indole derivatives; (2) Serotonin pathway: Peripheral production of the neurotransmitter serotonin by enterochromaffin cells is influenced by the gut microbiota, and the brain can also synthesize serotonin; (3) Kynurenine pathway: kynurenine-producing pathway plays a critical role in immune response and neurobiological functions, which is also affected by gut microbiota. (**f**) The ratios of KYN/TRP and KYNA/KYN at 7 days post-TBI. ↑, increased; ↓, decreased in 7 days post-TBI mice. The tryptophan and its metabolite listed in the pathways were marked with black (unchanged), red (increased), and blue (decreased) solid boxes. Data are shown as median with range. * *p* < 0.05, ** *p* < 0.01 *** *p* < 0.001 using unpaired Student’s *t* test. TBI, traumatic brain injury; TRP, tryptophan; IPYA, indole-3-pyruvic acid; ILA, indole-3-lactic acid; IA, indole acrylic acid; IAAld, indole-3-acetaldehyde; IEt, indole-3-ethanol (tryptophol); IAM, indole-3-acetamide; IAA, indole acetic acid; 5-HT, 5-hydroxytryptamine (serotonin); KYN, kynurenine; KYNA, kynurenic acid; XA, xanthurenic acid; ns, no significant.

**Figure 7:**
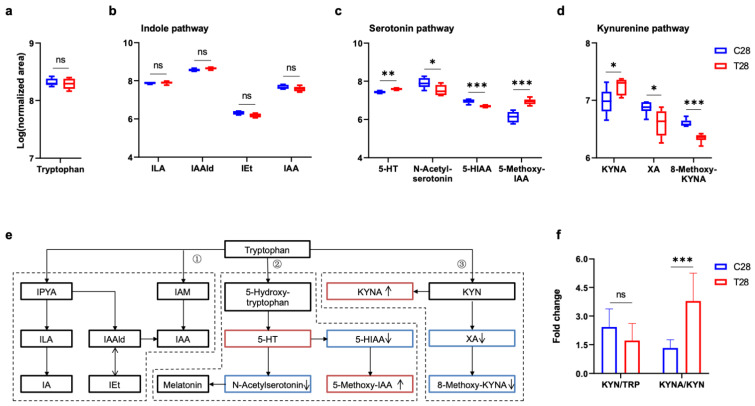
Alteration of serum tryptophan metabolic pathway at 28 days post-TBI. Comparison of serum tryptophan (**a**) and major tryptophan metabolites in indole pathway (**b**), serotonin pathway (**c**), and kynurenine pathway (**d**) at 28 days post-TBI. (**e**) Major tryptophan metabolism pathways are involved: (1) Indole pathway: Tryptophan can be directly metabolized by the gut microbiota into indole derivatives; (2) Serotonin pathway: Peripheral production of the neurotransmitter serotonin by enterochromaffin cells is influenced by the gut microbiota, and the brain can also synthesize serotonin; (3) Kynurenine pathway: Kynurenine-producing pathway plays a critical role in immune response and neurobiological functions, which is also affected by gut microbiota. (**f**) The ratios of KYN/TRP and KYNA/KYN at 28 days post-TBI. ↑, increased; ↓, decreased in 28 days post-TBI mice. The tryptophan and its metabolite listed in the pathways were marked with black (unchanged), red (increased), and blue (decreased) solid boxes. Data are shown as median with range. * *p* < 0.05, ** *p* < 0.01, *** *p* < 0.001 using unpaired Student’s *t* test. TBI, traumatic brain injury; TRP, tryptophan; IPYA, indole-3-pyruvic acid; ILA, indole-3-lactic acid; IA, indole acrylic acid; IAAld, indole-3-acetaldehyde; IEt, indole-3-ethanol (tryptophol); IAM, indole-3-acetamide; IAA, indole acetic acid; 5-HT, 5-hydroxytryptamine (serotonin); 5-HIAA, 5-hydroxyindole acetic acid; IAA, indole acetic acid; KYNA, kynurenic acid; XA, xanthurenic acid; ns, no significant.

In consideration of tryptophan co-metabolized by gut microbiota and host [12], and tryptophan metabolic balance influencing microglial activity [21], changes in various tryptophan metabolites produced from different metabolic pathways were focused at 7 days and 28 days post-TBI. Tryptophan is one of the essential amino acids only obtained from the daily diet, the serum level of which did not change post-TBI (Figure 6a and Figure 7a). Tryptophan can be directly metabolized by the gut microbiota into several molecules, such as indole and its derivatives [12]. Indole-3-acetaldehyde (IAAld) and indole-3-ethanol (IEt) were increased, and indole-3-lactic acid (ILA) and skatole were decreased at 7 days post-TBI (Figure 6b), whereas these changes in indole pathway were absent at 28 days post-TBI (Figure 7b). For the serotonin pathway, 5-hydroxytryptamine (5-HT) was increased at both 7 days and 28 days post-TBI (Figure 6c and Figure 7c). 5-HT as an important neurotransmitter triggers numerous functions in gut–brain signaling and brain physiology. Additionally, melatonin and 5-hydroxy indole acetic acid (5-HIAA) were reduced at 7 days and 28 days post-TBI, respectively. Tryptophan (TRP) metabolism through the kynurenine (KYN) pathways leads to the production of KYN and downstream products such as kynurenic acid (KYNA) and quinolinic acid [22]. KYNA is often considered to be neuroprotective [13], which was decreased at 7 days post-TBI (Figure 6d) but increased at 28 days post-TBI (Figure 7d). In addition, xanthurenic acid (XA) was decreased at both 7 days and 28 days post-TBI. All changes were shown in the pathway of TRP metabolism (Figure 6e and Figure 7e). The ratio of KYN to TRP was increased at 7 days post-TBI and returned to normal at 28 days post-TBI, while the ratio of KYNA to KYN was decreased at 7 days post-TBI and increased at 28 days post-TBI (Figure 6f and Figure 7f). The pattern of increased KYN to TRP but decreased KYNA to KYN ratio suggests an elevated metabolism down the neurotoxic pathway [13]. However, how these alterations of tryptophan metabolism controlled by gut microbiota play a role in the persistent upregulation of Lyz2 and microglial activation still needs further study.

### 3.4. Composition and Function of Gut Microbiota Progressively Shifted to Dysbiosis Post-TBI

**Figure 8:**
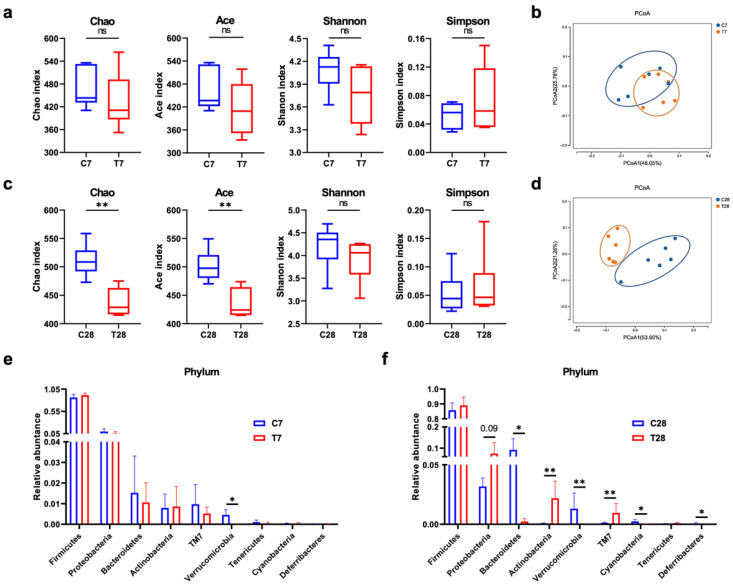
Compositional change of gut microbiota post-TBI. The α-diversity of the gut microbiota at 7 days (**a**) and 28 days (**b**) post-TBI (*n* = 6 per group). PCoA of the gut microbiota composition with weighted UniFrac distance at 7 days (**c**) and 28 days (**d**) post-TBI (*n* = 6 per group). Relative abundance of gut microbiota at the phylum level at 7 days (**e**) and 28 days (**f**) post-TBI (*n* = 6 per group). Data are shown as median with range. * *p*< 0.05, ** *p* < 0.01 using Wilcoxon rank-sum test. TBI, traumatic brain injury; PCoA, principal coordinate analysis; ns, no significant.

**Figure 9:**
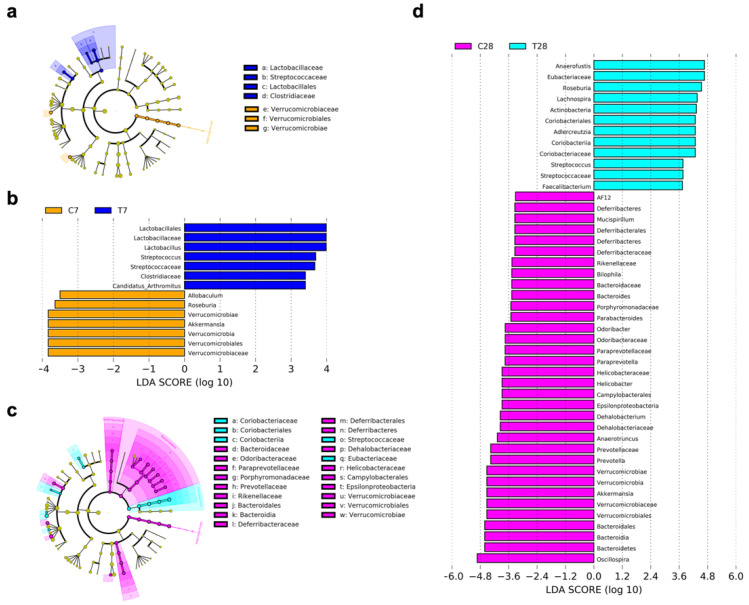
Linear discriminant analysis effect size of gut microbiota. Cladogram of LEfSe at 7 days (**a**) and 28 days (**c**) post-TBI. LDA score of LEfSe at 7 days (**b**) and 28 days (**d**) post-TBI.

The effects of antibiotics and FMT on microglia and Lyz2 indicated that the dysbiosis of the gut microbiota contributed to chronic neuroinflammation post-TBI. Therefore, the composition of the gut microbiota was identified by 16s rRNA gene sequencing to make sense of the possible mechanism. The α-diversity was expressed as Chao, Ace, Shannon, and Simpson indexes, which were unchanged at 7 days post-TBI (Figure 8a). Chao and Ace indexes were significantly decreased at 28 days post-TBI (Figure 8c). The β-diversity was measured using principal coordinates analysis (PCoA) with the weighted UniFrac distance, the change of which was more obvious at 28 days post-TBI (Figure 8b,d). At the phylum level, only the relative abundance of Verrucomicrobia was significantly decreased at 7 days post-TBI (Figure 8e). At 28 days post-TBI, in addition to Verrucomicrobia, the relative abundance of Bacteroidetes, Cyanobacteria, and Deferribacteres was also significantly decreased, with an increase in the relative abundance of Actinobacteria and TM7 (Figure 8f). Additionally, the linear discriminant analysis (LDA) effect size (LEfSe) showed that the taxa of differential bacteria were more at 28 days post-TBI than those at 7 days post-TBI (Figure 9). Specifically, *Streptococcus* belonging to *Streptococcaceae* was continually increased at 7 days and 28 days post-TBI, and meanwhile, *Akkermansia*, a beneficial bacterium belonging to Verrucomicrobia [23], was continually decreased at 7 days and 28 days post-TBI.

**Figure 10:**
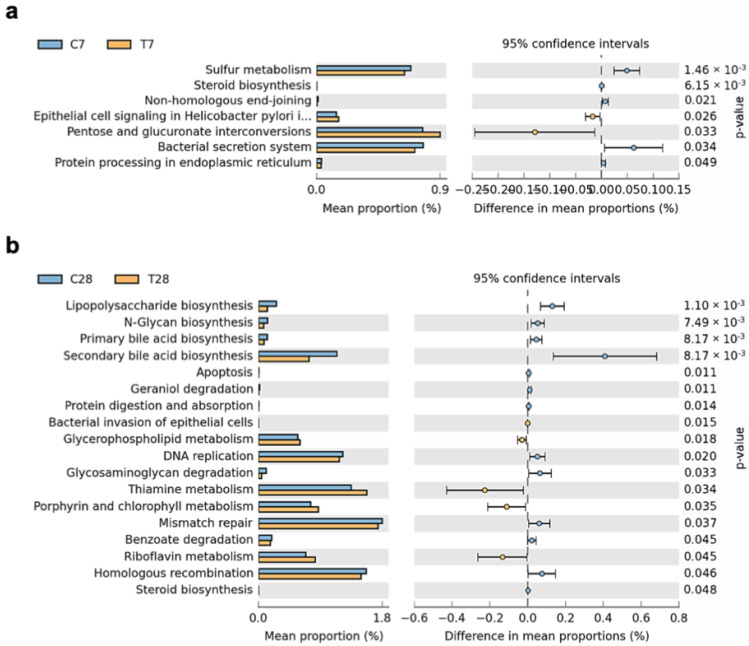
Functional difference analysis of gut microbiota. A comparison of the KEGG functional categories inferred from the 16S rRNA gene sequences using PICRUSt at 7 days (**a**) and 28 days (**b**) post-TBI. (*n* = 6 per group). KEGG, Kyoto Encyclopedia of Genes and Genomes; PICRUSt, phylogenetic investigation of communities by reconstruction of unobserved states.

Compositional changes will lead to altered gut microbiota function, therefore PICRUSt analysis was performed to assess the functional changes of the gut microbiota post-TBI at KEGG level 3. Sulfur metabolism, steroid biosynthesis, no-homologous end-joining, bacterial secretion system, and protein processing in the endoplasmic reticulum were proportionally decreased, and epithelial cell signaling in Helicobacter pylori infection and pentose and glucuronate interconversions were proportionally increased at 7 days post-TBI (Figure 10a). However, all these functional changes of gut microbiota at 7 days post-TBI were absent and new functional changes occurred at 28 days post-TBI. Gut microbiota functions of biosynthesis, including lipopolysaccharide, *n*-Glycan, primary bile acid, secondary bile acid, and steroid, were decreased at 28 days post-TBI, with an increase in the metabolism of glycerophospholipid, thiamine, porphyrin, chlorophyll, and riboflavin (Figure 10b). Taken together, the composition and function of gut microbiota post-TBI shifted over time, indicating that the changes in the gut microbial metabolism may be involved in the prolonged microglia activation and chronic neuroinflammation post-TBI.

## 4. Discussion

Traumatic brain injury (TBI) has been considered as a chronic and progressive disease with long-term consequences, but not a single static brain injury event [3,6,24]. A growing number of preclinical and clinical studies into the role of gut microbiota in the modulation of central nervous system (CNS) function have demonstrated that gut microbiota is involved in the regulation of complex cellular and molecular mechanisms, which are fundamental to the progressive pathophysiological processes of acute CNS injury, including TBI [8,25]. Therefore, the present study observed long-term changes in the composition and function of gut microbiota at 7 days and 28 days post-TBI, modifying gut microbiota by oral antibiotics and FMT to study its role in microglia activation, and further RNA sequencing and untargeted metabolome were used to explore the underlying mechanisms.

The composition of gut microbiota is dynamically changed post-TBI. In clinical research, the characteristics of gut microbiota were observed on days 0, 3, and 7 after admission in TBI patients, which were widely colonized by the Proteobacteria phylum, with *Enterobacteriaceae* forming the largest group [26]. In another small cohort study, the gut microbiota compositions of the patients with severe or moderate TBI significantly differed from that of the healthy volunteers, with an increased relative abundance of *Enterococcus*, *Parabacteroides*, *Akkermansia*, and *Lachnoclostridium*, and decreased *Bifidobacterium* and *Faecalibacterium* [27]. It was also reported that altered gut microbiota persisted for years post-TBI [28]. In the controlled cortical impact (CCI)-induced TBI mouse, *Lactobacillus gasseri*, *Ruminococcus flavefaciens*, and *Eubacterium ventriosum* were significantly decreased, while *Eubacterium sulci*, and *Marvinbryantia formatexigens* were significantly increased at 24 h post-TBI [29]. In addition, gut microbiota compositions determined from the fecal samples of pre-TBI and 2 h, 1, 3, and 7 days afterward suggested that TBI altered the composition of gut microbiota in a time-dependent manner [30]. In our study, significant changes in the gut microbiota were observed at 7 days and 28 days post-TBI. While there were varying trends in the composition of gut microbiota across time, some changes persisted through 28 days, such as a decreased relative abundance of *Akkermansia*, in the absence of therapeutic intervention. *Akkermansia muciniphila* is a paradigm for next-generation beneficial microorganisms [23], and a supplement may ameliorate the dysbiosis of gut microbiota and improve the outcome of TBI. Indeed, modifying gut microbiota affects the outcome of TBI. *Clostridium butyricum* and *Lactobacillus acidophilus* supplement had neuroprotective effects in TBI mice [31,32], whereas the antibiotic-induced dysbiosis of gut microbiota leads to neuroinflammation and impaired neurogenesis post-TBI [33]. However, although differences in gut microbiota composition have been observed in TBI patients and animal models, and the modulation of gut microbiota affects the outcome of TBI, the exact regulatory mechanism remains elusive.

The function of gut microbiota in the host is often mediated by its metabolites [20]. Bile acids, short-chain fatty acids (SCFAs), and tryptophan metabolites are three major groups of the most studied gut microbiota-related metabolites in health and disease [34]. In a retrospective clinical analysis, serum bile acid level was greatly reduced in TBI patients [35]. A similar alteration of the bile acid profile was also observed in TBI mice. Bile acids were decreased in feces and plasm post-TBI, with a more significant decrease in secondary bile acids, which was associated with specific bacterial taxa [36]. A reduction in total SCFAs was detected in fecal samples at 24 h and 28 days post-TBI, and the supplementation of SCFAs improved spatial learning post-TBI [37].

TBI was also accompanied by altered tryptophan metabolism. In the present study, untargeted UPLC-MS revealed that serum tryptophan metabolism was significantly changed at 7 days as well as 28 days post-TBI. The indole pathway of tryptophan metabolism was changed at 7 days post-TBI and returned to normal at 28 days post-TBI. A microbial indole metabolite, indole-3-propionic acid (IPA), effectively alleviated neuroinflammation, neurological impairment, and brain infarction in mice with ischemic brain injury [38]. For neurodegenerative diseases, IPA had neuroprotective properties against the Alzheimer beta-amyloid [39]. IPA-induced aryl hydrocarbon receptor (AhR) activation inhibited neuroinflammation in APP/PS1 mice, a mouse model of Alzheimer’s disease (AD) [40]. Indole-3-acetaldehyde (IAAld), also a ligand of AhR, was increased at 7 days post-TBI, which might be involved in the regulation of neuroinflammation. Additionally, IPA could be used as a biomarker to predict the prognosis of TBI, AD, and Parkinson’s disease [41,42,43]. In peripheral nervous injury, IPA enhanced axonal regeneration and accelerated the recovery of sensory function by inhibition of neutrophil chemotaxis [44]. However, IPA was not detected in our study, which might be due to the time point of sample collation or the limitation of untargeted UPLC-MS. The long-term elevation of serotonin was observed in this study, but its role in the TBI is still unclear. Melatonin, a downstream product of serotonin, alleviated gut dysbiosis and neuroinflammation [45,46], which was decreased at 7 days post-TBI in our study. The kynurenine pathway of tryptophan metabolism produces several neuroactive metabolites, including neurotoxic quinolinic acid and neuroprotective kynurenic acid (KYNA) [13]. An imbalance in the kynurenine pathway is a potential molecular pathway by which the TBI-induced neurometabolic cascade may contribute to the development of chronic neuroinflammatory response [13]. Kynurenine significantly increased at 21 days post-TBI, and a significant decrease in the ratio of serotonin to tryptophan and the ratio of melatonin to tryptophan was noted at 21 days post-TBI [47]. We found that kynurenine was significantly increased and KYNA was decreased at 7 days post-TBI, but kynurenine was unchanged and KYNA was increased at 28 days post-TBI, with persistent reductions in xanthurenic acid (XA) and 8-methoxy-kynurenic acid. The ratio of plasma kynurenine to tryptophan reflects systemic indoleamine 2,3-dioxygenase (IDO) activity, which is inducible under pro-inflammatory and plays a pivotal role in shaping immune tolerance [48]. The pattern of increased IDO activity (kynurenine to tryptophan ratio) and decreased kynurenine aminotransferase enzyme (KAT) activity (KYNA-to-kynurenine ratio) suggests elevated metabolism down the neurotoxic pathway [13]. Additionally, the uptake and metabolism of tryptophan in activated microglia lead to the production of neurotoxic metabolites, such as 3-hydroxy-kynurenine (3HK) and quinolinic acid [13], although neither were not detected in our study. However, tryptophan metabolism is a complex process because the alteration of one of its three metabolic pathways will affect the others, and therefore consequences for all of its three metabolic pathways should be taken into consideration when studying its role in perpetual microglia activation and chronic neuroinflammation post-TBI.

Microglia plays a critical role in neuroinflammation post-TBI. The inflammatory response post-TBI is crucial to clearance of debris, repair, and regeneration, but dysregulated inflammation can contribute to additional acute and chronic brain injury [6]. After brain injury, microglia can produce neuroprotective factors, clean up debris, and coordinate neurorestorative processes, which contribute to neurological recovery [5]. However, dysregulated microglia can produce pro-inflammatory mediators inhibiting CNS repair and leading to neuronal dysfunction, which is dependent on their functional responses and polarization state post-TBI [5]. Additionally, persistent microglial activation and neuroinflammation, lasting many years in some cases of TBI, can cause chronic neurodegeneration, dementia, and encephalopathy [6]. Microglia-mediated mechanisms contributed to synaptic loss and cognitive impairment post-TBI [49]. However, the mechanism of persistent microglial activation post-TBI is still unclear. Growing evidence suggests that the gut microbiota can signal the brain to modulate CNS processes such as neurotransmission, neurogenesis, and microglial activation in the context of both homeostatic conditions and in response to stressors [9]. FMT derived from AD mice with gut microbiota dysbiosis increased activated microglia, aggravated neuroinflammatory response, and worsened neurological outcomes of TBI mice [50]. Therefore, the persistent dysbiosis of gut microbiota post-TBI may be responsible for the prolonged activation of microglia. In the present study, gut microbiota dysbiosis was observed at 7 days and 28 days post-TBI, which was more severe at 28 days post-TBI, suggesting that gut microbiota dysbiosis is progressive post-TBI. In addition, this progressive dysbiosis was along with persistent microglial activation. The depletion of gut microbiota by oral antibiotics suppressed microglial activation at 28 days post-TBI, but not at 7 days post-TBI, indicating that persistent microglial activation post-TBI is dependent on the progressive dysbiosis of gut microbiota, which was further supported by that FMT from TBI mice for 28 days, but not for 7 days, activated microglia of normal mice. Moreover, RNA sequencing identified a microglial activation gene, Lyz2, and its persisting upregulation post-TBI, which was also influenced by antibiotic administration and FMT, evidencing that gut microbiota-controlled Lyz2 upregulation may be responsible for microglia activation.

However, this study had a few limitations, which would be overcome in future research. One microglia marker was evaluated in the study, but additional markers, such as TMEM119, or flow cytometry were appreciated to be used to evaluate the dynamics of microglia phenotypes post-TBI. The effect of gut microbiota, instead of the gut microbiome, was discussed, and this model should be taken into consideration whether fungi and viruses have an impact. Additionally, based on the results of 16s rRNA sequencing, if changes in candidate bacteria, such as *Akkermansia* or others, were validated by PCR, the supplements of them could be used to study their influence on the microglia activation post-TBI. In addition, a targeted approach was preferable for metabolite confirmation. Furthermore, using a gene knockout animal was an ideal method to clarify the role of Lyz2 in microglia activation post-TBI. Finally, the regional specialization of microglia in the brain might be studied deeply in the chronic neuroinflammation post-TBI.

In conclusion, TBI-induced gut microbiota progressive dysbiosis may contribute to persistent Lyz2 upregulation via altering tryptophan metabolic phenotype, resulting in perpetual microglia activation and chronic neuroinflammation. Further studies of the precise mechanistic pathways are warranted.

## Data Availability

All sequencing data were deposited in the National Center for Biotechnology In-formation (NCBI) database with BioProject ID: PRJNA851721 (16s rRNA gene sequencing) and PRJNA855975 (RNA sequencing). Further inquiries can be directed to the corresponding authors.
